# External Validation of an EHR-Based Model for Risk of Patient No-Show in Primary Care

**DOI:** 10.1001/jamanetworkopen.2025.21637

**Published:** 2025-07-17

**Authors:** Afiba Manza-A. Agovi, Mirsada Serdarevic, Aaron W. Gehr, Caitlin T. Thompson, Wentao Li, Jeff Claassen, Matthew Cvitanovich, Rohit P. Ojha

**Affiliations:** 1 Center for Epidemiology & Healthcare Delivery Research, JPS Health Network, Fort Worth, Texas; 2 Department of Quantitative and Qualitative Health Sciences, UT Health San Antonio, San Antonio, Texas; 3 Cook Children’s Health Care System, Fort Worth, Texas

## Abstract

This prognostic study evaluates the performance of an electronic health record (EHR)–based model for predicting nonattendance at primary care appointments overall and by race and ethnicity.

## Introduction

Nonattendance at primary care appointments (ie, no-shows) negatively impacts health care organizations and patients, leading to inefficient use of resources, financial losses, and suboptimal outcomes, among other consequences.^1,2^ Prediction models^3^ are a promising strategy to identify patients with a high risk of no-show to prioritize for targeted outreach.^4^

The proprietary Risk of Patient No-Show Model^5,6^ (Epic Systems) is used in more than 142 health care organizations. Nevertheless, limited evidence is available about external validity, which raises concerns about potentially misallocated resources and inadequate patient care. Therefore, we aimed to evaluate overall and race and ethnicity–specific performance of the first version of the model among primary care patients.

## Methods

This prognostic study follows the TRIPOD + AI reporting guidelines. The North Texas Regional institutional review board approved this study with a waiver of informed consent given that the study uses secondary data and posed minimal risk to patients. We used electronic health record (EHR) data from 12 community health clinics within JPS Health Network, an urban safety-net health system in north Texas. Eligible patients were aged 18 years and older and scheduled an appointment between May 1, 2022, and April 30, 2023. We used the first scheduled appointment within the study period and excluded walk-in and same-day appointments, as recommended by the model’s developer.^5^

We used predictor definitions specified by the developer^5^ and obtained model-based predictions automatically generated from scheduling workflows within the EHR. Our outcome was final appointment status, defined as no-show vs all other statuses, including completed, partial visit, left without being seen, and canceled.

We assessed overall and race and ethnicity–specific area under the receiver operating characteristic curve (AUC), mean calibration, calibration-in-the-large, calibration slope, and net benefit.^3^ Race and ethnicity were self-reported and used to assess model fairness. We also explored the sensitivity of model performance to changes in eligibility criteria and outcome definition. The eMethods in Supplement 1 includes additional details about the model and analysis. We used Stata version 18.1 (StataCorp) and R version 4.4.1 (R Project for Statistical Computing) for the analyses.

## Results

Our eligible population comprised 92 457 patients. The median (IQR) age was 50 (36-61) years, 60.9% were female, 33.7% were Hispanic, 30.5% were non-Hispanic Black, 23.2% were non-Hispanic White, and 12.6% were non-Hispanic and belonged to an additional racial group; 42.9% were self-pay or charity care program beneficiaries. Overall, 23.0% of appointments were no-show.

The Table summarizes overall and race and ethnicity–specific performance measures. Overall, the AUC was 0.58, mean calibration was 0.99, calibration-in-the-large was 0.02, and calibration slope was 0.41. Race and ethnicity–specific performance was similar, except the AUC was 0.53 and calibration slope was 0.18 for non-Hispanic patients from additional racial groups; mean calibration was less than 1.00 and calibration-in-the-large was greater than 0 for non-Hispanic Black patients. The Figure illustrates greater net benefit of the model overall and for racial and ethnic groups than treat-all or treat-none strategies at some decision thresholds between 20% and 30%.

**Table.** Overall and Race and Ethnicity–Specific Discrimination and Calibration Metrics of the No-Show Risk Model

Group	Estimate (95% CI)			
	AUC	Mean calibration	Calibration-in-the-large	Calibration slope
Overall	0.58 (0.58 to 0.59)	0.99 (0.98 to 1.00)	0.02 (0.00 to 0.03)	0.41 (0.39 to 0.43)
Race/ethnicity				
Hispanic	0.58 (0.57 to 0.59)	1.01 (0.99 to 1.03)	−0.01 (−0.04 to 0.02)	0.41 (0.38 to 0.45)
Non-Hispanic Black	0.58 (0.57 to 0.59)	0.86 (0.84 to 0.88)	0.23 (0.20 to 0.25)	0.41 (0.38 to 0.45)
Non-Hispanic White	0.60 (0.59 to 0.61)	1.05 (1.02 to 1.09)	−0.07 (−0.11 to −0.04)	0.52 (0.48 to 0.57)
Non-Hispanic, additional groups^a^	0.53 (0.52 to 0.55)	1.27 (1.22 to 1.33)	−0.32 (−0.38 to −0.28)	0.18 (0.11 to 0.24)

Abbreviation, AUC, area under the receiver operating characteristic curve.

^a^ Includes American Indian or Alaska Native individuals, Asian individuals, Native Hawaiian or Other Pacific Islander individuals, individuals with combinations of American Indian or Alaska Native, Asian, and Native Hawaiian or Other Pacific Islander race, and no race and ethnicity information provided.

**Figure.**
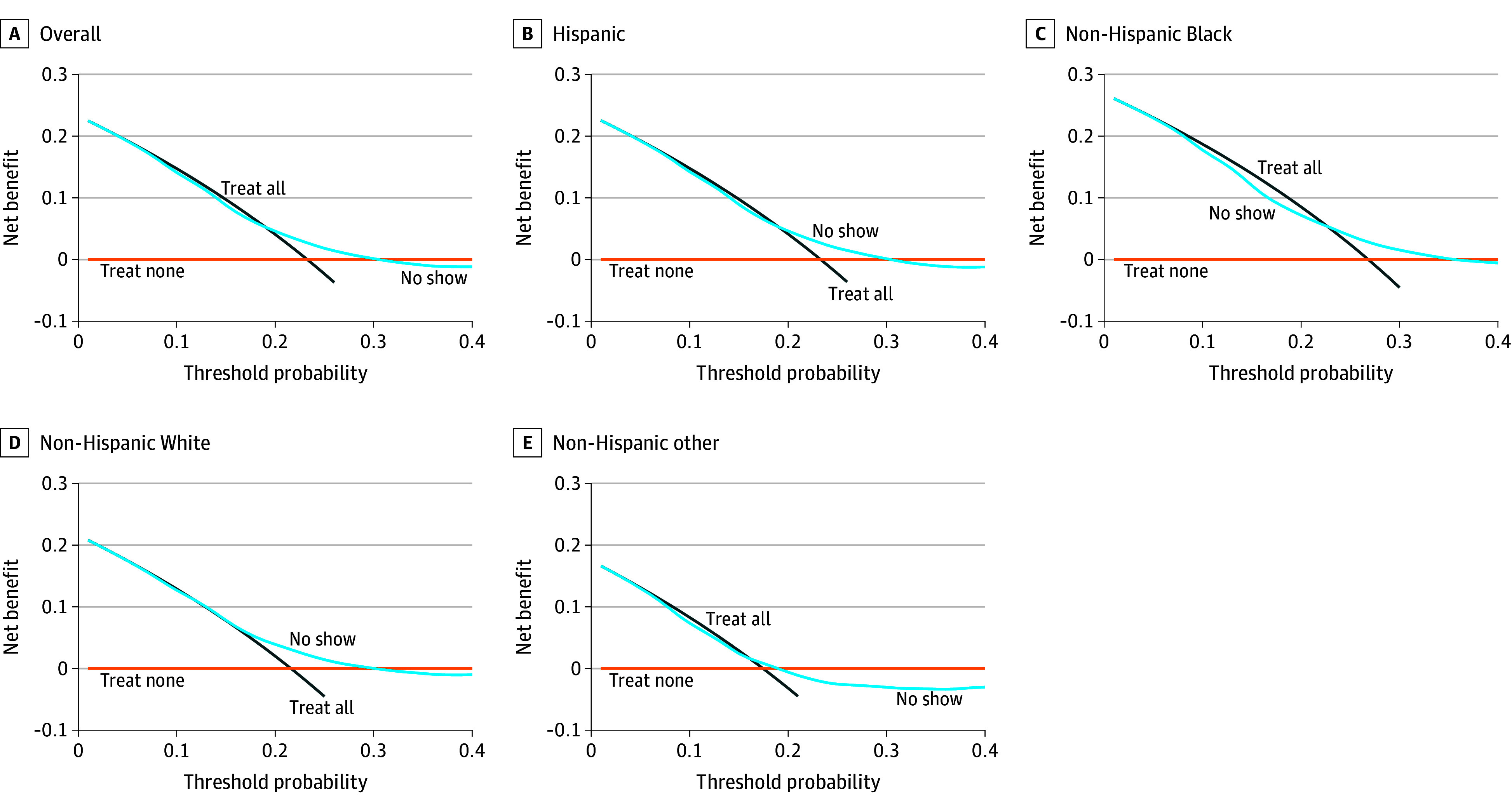
Overall and Race and Ethnicity–Specific Net Benefit of the No-Show Risk Model Decision curve analysis illustrating the net benefit of the no-show risk model overall and stratified by race and ethnicity, compared with treat all or treat none strategies. The model had modest net benefit at decision thresholds between 20% and 30% in the study population.

Our sensitivity analyses indicated that use of the model’s eligibility criteria or its second version’s outcome definition improved discrimination but worsened calibration. Net benefit of the model shifted to the 30% to 45% range when we used the model’s eligibility criteria and was entirely negated when we used the second version’s outcome definition.

## Discussion

In this study, the no-show risk model had limited discrimination. Despite respectable mean calibration, the predicted probabilities for no-show were overestimated for high-risk patients and underestimated for low-risk patients. Nevertheless, the model had modest clinical utility compared with outreach for all or no outreach strategies if the decision threshold was between 20% and 30% in our population.

We were limited to a single predicted no-show probability, which ranged between 3 and 7 days prior to the appointment. Our results thus represent an aggregate of different time horizons. Ideally, no-show probabilities would be generated at a uniform time horizon consistent with the intended moment of use. In addition, our findings may not generalize to all health care settings.

A risk-based outreach strategy using this model within the beneficial range of decision thresholds could substantially reduce the number of patients for outreach, which would reduce burden on operational staff. Nevertheless, health care organizations will need to consider the potential trade-off of missed outreach opportunities for patients belonging to some racial and ethnic groups, depending on the decision threshold.

## Notes

Supplement 1.
eMethods.

eReferences.

Supplement 2.
Data Sharing Statement
