# Supplementary material for: External Validation of an EHR-Based Model for Risk of Patient No-Show in Primary Care
Source: JAMA Netw Open. 2025 Jul 17;8(7):e2521637. doi: 10.1001/jamanetworkopen.2025.21637 (PMC12272289; doi:10.1001/jamanetworkopen.2025.21637)
Supplement: Supplement 1. — eMethods. eReferences. [file jamanetwopen-e2521637-s001.pdf]

# Supplemental Online Content

Agovi AMA, Serdarevic M, Gehr AW, et al. External validation of an EHR-based risk of patient no-show model in primary care. *JAMA Netw Open.* 2025;8(7):e2521637. doi:10.1001/jamanetworkopen.2025.21637

**eMethods.**

**eReferences.**

This supplemental material has been provided by the authors to give readers additional information about their work.

© 2025 Agovi AMA et al. *JAMA Network Open*.

**eMethods**

*The Epic No-show Model*

The Epic Risk of Patient No-Show Model (Version 1) is a Naïve Bayes machine learning model, which uses local historical data (i.e., from the health system) to predict the probability of no-show.[1] For training on local data, the model includes only appointments with a status of "Completed" or "No Show" and excludes walk-ins. The model uses predictors including patient characteristics, appointment characteristics, and appointment history. As of May 2022, updates from Epic Systems removed all demographic predictors from the model to avoid potential exacerbation of inequities for marginalized populations.[1]

We obtained model predictions from scheduling workflows within the electronic health record (EHR), which were automatically generated based on combinations of patient- and appointment-level predictors included in the Epic Risk of Patient No-Show Model (Version 1).[1] Only the first scheduled appointment was considered to ensure each patient had only one scheduled appointment. Model predictors[1] included marital status; financial class at the time of scheduling; preferred language; last recorded body mass index; smoking status; the number of no-shows in the two most recent appointments; month, day of the week, and hour of the appointment; department specialty; lead time (time between scheduling and the appointment); and binary indicators for whether the appointment was modified or confirmed, if the appointment was with a primary care provider, and if a referral was required or already in place. Consistent with the original model, missing values were imputed based on the type of predictor. Specifically, binary predictors were set to 0, categorical predictors were set to the most frequently observed, and numeric values were set to the sample mean.[1] Despite the availability of model-based predicted probabilities in our EHR, the model is not currently used for decision-

© 2025 Agovi AMA et al. *JAMA Network Open*.

making in our organization. This validation study was intended to generate evidence about whether the model could be used for decision-making.

*Data Analysis*

The Epic Risk of Patient No-show Model automatically generates predicted probabilities for each appointment, which were extracted from Epic Clarity and subsequently used to assess discrimination, calibration, and net benefit of the model. We assessed discrimination using the area under the receiver operating characteristic curve (AUC). The AUC ranges from 0.50 to 1.0, where larger values indicate that predicted probabilities for cases (i.e., appointments with a no-show status) are larger than for non-cases (i.e., any other appointment status).[2,3] We assessed calibration using graphical and empirical measures.[4,5] We plotted calibration curves based on expected and observed risks of no-show for graphical evaluation of model calibration.[6] We estimated mean calibration and calibration-in-the-large, which assess over- or under-estimation in the population. We estimated calibration slope to assess whether the predicted probabilities were too extreme or not extreme enough.[4,7] We assessed the net benefit of the Epic Risk of Patient No-show Model using decision curve analysis, which visualizes the clinical utility of a model across a range of decision thresholds compared with intervening on no one or intervening on everyone for no-show prevention. Epic Systems did not recommend a pre-specified decision threshold for the model. Therefore, we used thresholds (15%, 30%, and 40%) reported in prior studies to inform our assessment.[8-10] Lastly, we repeated these analyses within racial/ethnic subgroups to assess potential variation in model performance across groups for insights about equitable utility.

*Sensitivity Analysis*

© 2025 Agovi AMA et al. *JAMA Network Open*.

Our eligibility criteria differed from the original criteria used for developing the Epic Risk of Patient No-show Model to better reflect use in our setting. We included patients with completed, partial visit, left without being seen, and canceled appointment statuses, whereas the original criteria included only completed appointments. In addition, we used Version 1 of the model because Version 2 was unavailable in our Epic environment. A key difference is that Version 2 includes canceled appointments as part of the outcome definition. Therefore, we explored the sensitivity of model performance to using the original eligibility criteria and the outcome definition from Version 2 of the model. We used the same methods for assessing discrimination, calibration, and net benefit as the main analysis.

© 2025 Agovi AMA et al. *JAMA Network Open*.

**eReferences**


1. Epic Systems. Cognitive Computing Brief: Risk of Patient No-Show (Version 1). 2022.
2. Hanley JA, McNeil BJ. The meaning and use of the area under a receiver operating characteristic (ROC) curve. Research Support, Non-U.S. Gov't. *Radiology*. Apr 1982;143(1):29-36. doi:10.1148/radiology.143.1.7063747
3. Cook NR. Use and misuse of the receiver operating characteristic curve in risk prediction. Research Support, N.I.H., Extramural Research Support, Non-U.S. Gov't. *Circulation*. Feb 20 2007;115(7):928-35. doi:10.1161/CIRCULATIONAHA.106.672402
4. Van Calster B, McLernon DJ, van Smeden M, et al. Calibration: the Achilles heel of predictive analytics. *BMC Med*. Dec 16 2019;17(1):230. doi:10.1186/s12916-019-1466-7
5. Van Calster B, Nieboer D, Vergouwe Y, De Cock B, Pencina MJ, Steyerberg EW. A calibration hierarchy for risk models was defined: from utopia to empirical data. *J Clin Epidemiol*. Jun 2016;74:167-76. doi:10.1016/j.jclinepi.2015.12.005
6. Austin PC, Steyerberg EW. Graphical assessment of internal and external calibration of logistic regression models by using loess smoothers. Research Support, Non-U.S. Gov't. *Stat Med*. Feb 10 2014;33(3):517-35. doi:10.1002/sim.5941
7. Steyerberg EW. *Clinical Prediction Models*. 2nd ed. Statistics for Biology and Health. Springer Nature Switzerland AG; 2019.
8. Tarabichi Y, Higginbotham J, Riley N, Kaelber DC, Watts B. Reducing Disparities in No Show Rates Using Predictive Model-Driven Live Appointment Reminders for At-Risk Patients: a Randomized Controlled Quality Improvement Initiative. *J Gen Intern Med*. Oct 2023;38(13):2921-2927. doi:10.1007/s11606-023-08209-0
9. Ulloa-Perez E, Blasi PR, Westbrook EO, Lozano P, Coleman KF, Coley RY. Pragmatic Randomized Study of Targeted Text Message Reminders to Reduce Missed Clinic Visits. *Perm J*. Apr 5 2022;26(1):64-72. doi:10.7812/TPP/21.078
10. Rojas J. C. BKG, Fahrenbach J, Selling M. K., Shah S, Zhou Z,. External validation of the proprietary Epic no-show prediction model at a single academic medical center. American Journal of Respiratory and Critical Care Medicine; 2023:A1421.


© 2025 Agovi AMA et al. *JAMA Network Open*.
