# Supplementary material for: External Validation of an EHR-Based Model for Risk of Patient No-Show in Primary Care
Source: JAMA Netw Open. 2025 Jul 17;8(7):e2521637. doi: 10.1001/jamanetworkopen.2025.21637 (PMC12272289; doi:10.1001/jamanetworkopen.2025.21637)
Supplement: Supplement 2. — Data Sharing Statement [file jamanetwopen-e2521637-s002.pdf]

Data Sharing Statement

Agovi. External Validation of an EHR-Based Risk of Patient No-Show Model in Primary Care. *JAMA Netw Open*. Published July 17, 2025. doi:10.1001/jamanetworkopen.2025.21637

Data
Data available: Yes
Data types: Deidentified participant data
How to access data: The data for this study are available on reasonable request to the corresponding author and review by the JPS Health Network External Data Governance Committee (research@jpshealth.org)
When available: With publication

Supporting Documents
Document types: None

Additional Information
Who can access the data: The data for this study are available on reasonable request to the corresponding author and review by the JPS Health Network External Data Governance Committee (research@jpshealth.org)
Types of analyses: For a specified purpose
Mechanisms of data availability: The data for this study are available on reasonable request to the corresponding author and review by the JPS Health Network External Data Governance Committee (research@jpshealth.org)
